# Arterial pulse attenuation prediction using the decaying rate of a pressure wave in a viscoelastic material model

**DOI:** 10.1007/s10237-017-0980-9

**Published:** 2017-11-22

**Authors:** J. Menacho, L. Rotllant, J. J. Molins, G. Reyes, A. A. García-Granada, M. Balcells, J. Martorell

**Affiliations:** 1 IQS School of Engineering, Universitat Ramon Llull, Via Augusta 390, 08017 Barcelona, Spain; 2 IMES – MIT, 77 Massachusetts Av., E25-229, Cambridge, MA 02139 USA; 3 Department of Applied Sciences, CBSET, 500 Shire Way, Lexington, MA USA

**Keywords:** Pressure wave damping, Circulatory system, Cardiovascular disease, Computational fluid dynamics

## Abstract

The present study examines the possibility of attenuating blood pulses by means of introducing prosthetic viscoelastic materials able to absorb energy and damp such pulses. Vascular prostheses made of polymeric materials modify the mechanical properties of blood vessels. The effect of these materials on the blood pulse propagation remains to be fully understood. Several materials for medical applications, such as medical polydimethylsiloxane or polytetrafluoroethylene, show viscoelastic behavior, modifying the original vessel stiffness and affecting the propagation of blood pulses. This study focuses on the propagation of pressure waves along a pipe with viscoelastic materials using the Maxwell and the Zener models. An expression of exponential decay has been obtained for the Maxwell material model and also for low viscous coefficient values in the Zener model. For relatively high values of the viscous term in the Zener model, the steepest part of the pulse can be damped quickly, leaving a smooth, slowly decaying wave. These mathematical models are critical to tailor those materials used in cardiovascular implants to the mechanical environment they are confronted with to repair or improve blood vessel function.

**Electronic supplementary material:**

The online version of this article (10.1007/s10237-017-0980-9) contains supplementary material, which is available to authorized users.

## Introduction

Recent evidence suggests a link between cardiovascular and neurodegenerative diseases. Indeed, changes in pulsatile shear stress provoked by arterial stiffening inherent to the aging process, or indirect flow effects on the immunological system, can extend to the brain (Benetos et al. 1993; Martorell et al. 2012, 2014; Garcia-Polite et al. 2016), an organ with low peripheral resistance. This problem is common in hydraulic engineering, where pressure surges are damped by means of additional pipes or viscoelastic materials (Pezzinga and Scandura 1995; Pezzinga 2002; Covas et al. 2004). Herein, we hypothesize that a polymeric prosthesis implanted in a stiffened major vessel such as the aorta or the carotid could attenuate blood pulses. The viscoelastic properties of the material should absorb pulsatile energy and smooth such pulses to prevent brain damage. Several materials with medical applications, like polydimethylsiloxane (PDMS, silicone) or polytetrafluoroethylene (PTFE), show viscoelastic behavior (Calvo Aguilar 2013; Mahomed et al. 2015). These materials, widely used in many medical devices, vary the vessel stiffness and affect the propagation of blood pulses. As a first step, the study is focused on evaluating the damping rate of a pulse moving along a vessel of viscoelastic material. The aim of this manuscript is to study the mathematical model of a pressure wave propagating along a viscoelastic tube for Maxwell-type material models. This paper’s novelty resides in the design of prostheses able to damp changes in pulsatile flow which may jeopardize the brain microvasculature (Garcia-Polite et al. 2016).

Mathematical and computational models of the blood circulation are nowadays a topic of hard debate and scrutiny due to the myriad of conditions and pathologies associated with cardiovascular disease. The Windkessel model is a classic one (Keener and Sneyd 2009; Manning et al. 2002; Segers et al. 2003; Olufsen and Nadim 2004; Ellwein et al. 2008). Windkessel-type models account for the impedance to blood flow of the whole or of a part of the circulatory system. This reduces the whole vessel to a single-point equation and does not allow studying the damping phenomena that occurs along the vessel. To study the attenuation of a pulse, a pointwise model becomes necessary. One-dimensional models have also been extensively used to study pressure and flow wave propagation in the arteries (Olufsen et al. 2000; Alastruey et al. 2009; Qureshi et al. 2014; Willemet and Alastruey 2015; Alfonso et al. 2016), even coupled to the cerebrospinal fluid system (Martin et al. 2012). These models assume an elastic behavior of the vessels’ walls; therefore, the pressure is considered proportional to the cross-section area of the vessel with no viscoelastic component.

Propagation of a pressure wave in a filled pipe is a classic topic in engineering, with different applications, like water hammer or nondestructive testing of pipelines (Jiang et al. 2011; Liu et al. 2013; Meniconi et al. 2012). When the pipe material is elastic (and the friction effects negligible), the mathematical model leads to the wave equation. Viscoelastic materials characterization is not a simple task, and many mathematical models describe different phenomena like creep, relaxation and recovery. Modeling the viscoelastic properties of the arterial wall is not straightforward. There are a number of studies that consider different viscoelastic behaviors of the material of the vessel (Erbay et al. 1992; Demiray 1997; Kudryashov and Chernyavskii 2006; Guala et al. 2015), including fractional elements (Pérez Zerpa et al. 2015; Giusti and Mainardi 2016). A general approach using a Hook’s Law corrected with a quadratic term was deeply studied by Kudryashov and Chernyavskii (2006). Some works (Pezzinga et al. 2014, 2016) present quasi 2-D models, to take into account the viscous friction in the transients. Differential constitutive models, which present linearity, are a classic choice for studying a viscoelastic prosthesis (Drozdov 1996; Bergström and Hilbert 2005). Among them, the Kelvin–Voigt material model has been thoroughly studied and is broadly used in several engineering fields (Meniconi et al. 2012; Apollonio et al. 2014; Kundu et al. 2015; Warda et al. 2001; Barclay and Moodie 1987; Moodie et al. 1985). Unlike Kelvin–Voigt model, Maxwell and Zener models can simulate materials that show permanent deformation (Covas et al. 2004) and can be adjusted to the mechanical properties of vascular prostheses (Blaise et al. 2016). In this work, we have explored Maxwell and Zener models to study pressure waves’ attenuation when traveling along viscoelastic pipes. The final outcome of this study is explicit formulae that quantify the decaying rate of pressure waves in vascular viscoelastic prostheses. To the best of our knowledge, there is not such explicit formula for this class of materials. We will exploit these formulae to determine the material properties that fulfill a desired level of attenuation. This will be useful to choose commercially available materials or design and manufacture our own material to design a prosthesis that suits the patient’s attenuation needs.

## Materials and methods

In this manuscript, we model the transmission of pressure pulses along a straight pipe made of a viscoelastic material, filled with a fluid. This model can be separated in two parts: The first one refers to the dynamic stress/strain behavior of the material of the pipe, also known as material model, and the other one refers to the wave equation governing the pressure wave transmission. We have compared this model to a set of experimental strain/stress and strain/time curves and adjusted the parameters for a polymer for medical devices, polydimethylsiloxane.

### Material model

**Fig. 1:**
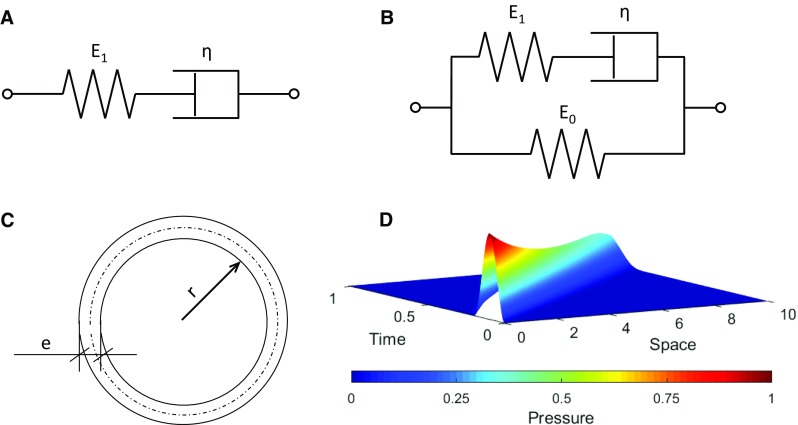
**a** Maxwell model for a viscoelastic material. E$_{1}$ is the elastic modulus and $\eta$ the viscous modulus. **b** Zener model for a viscoelastic material. E$_{0}$ and ${E}_{1}$ are the elastic moduli and $\eta$ the viscous modulus. **c** Sketch of the pipe section. r is the radius, and e is the thickness of the pipe. **d** One-dimensional pressure pulse propagation along a pipe in time and space

We have used two well-known viscoelastic material model, the Maxwell model (Fig. 1a) and the Zener model (Fig. 1b). The Maxwell model is defined by Eq. (1), where $\varepsilon$ is the strain and $\sigma$ the stress. $E_{1}$ and $\eta$ are the static Young modulus and the viscous factor, respectively. The Zener model (also known as standard linear viscoelastic model or three-parameter model) is defined by Eq. (2), where $\tau =\eta /E_1$.

1 $$\begin{aligned}&\frac{\partial \varepsilon }{\partial t}=\frac{1}{E_1 }\cdot \frac{\partial \sigma }{\partial t}+\frac{\sigma }{\eta } \end{aligned}$$

2 $$\begin{aligned}&\frac{\partial \sigma }{\partial t}+\frac{\sigma }{\tau }=(E_0 +E_1 ) \frac{\partial \varepsilon }{\partial t}+\frac{E_0 }{\tau }\varepsilon \end{aligned}$$

### Wave equation governing the pressure wave transmission

The hypotheses assumed for the model are that the fluid is incompressible; the effect of the viscosity of the fluid is negligible; and the deformation of the tube is small related to its radius. Fluid movement is a plug flow, so the velocity does not depend on the radial dimension. In fact, a published study in which speed was locally measured (Pezzinga et al. 2014) shows fairly flat velocity profiles in the hydraulic transients. However, it should be noted that this study was carried out for turbulent regimes, whereas flow is usually laminar in the circulatory system.

For a pipe with wall thickness (*e*) small compared to the inner radius (*r*), the relationship between the pressure difference between the pressure inside and outside the pipe (*p*) and the tangential stress ($\sigma$) at the pipe wall (Fig. 1c) is:

3 $$\begin{aligned} p\cdot r=\sigma \cdot e \end{aligned}$$

The continuity equation for a differential volume inside the tube gives:

4 $$\begin{aligned} \frac{\partial u}{\partial x}=-\frac{2}{r}\cdot \frac{\partial r}{\partial t} \end{aligned}$$

In addition, the balance of the momentum gives:

5 $$\begin{aligned} \rho \left( {\frac{\partial u}{\partial t}+u\frac{\partial u}{\partial x}} \right) =-\frac{\partial p}{\partial x}+\frac{f}{\pi r^{2}} \end{aligned}$$

where *u* is the velocity of the fluid and $\rho$ its density. In the last term, *f*is the frictional force per unit length that depends on the viscosity and the shape of the velocity (Willemet and Alastruey 2015). The term of convective acceleration may be negligible when the velocity is low. In this work, the convective acceleration and frictional force terms have been neglected.

### Pressure propagation using the Maxwell model

If the strain is $d\varepsilon =dr/r$, combining Eq. (1) with Eq. (4) gives:

6 $$\begin{aligned} \frac{\partial u}{\partial x}=-2\left( {\frac{1}{E_1 }\cdot \frac{\partial \sigma }{\partial t}+\frac{1}{\eta }\sigma } \right) \end{aligned}$$

Therefore, Eqs. (5, 6), after cross-derivation, give the unique equation:

7 $$\begin{aligned} \frac{\partial ^{2}p}{\partial x^{2}}=2\rho \left( {\frac{1}{E_1 }\cdot \frac{\partial ^{2}\sigma }{\partial t^{2}}+\frac{1}{\eta }\cdot \frac{\partial \sigma }{\partial t}} \right) \end{aligned}$$

Taking into account (3), if *c* is the pressure wave velocity, the model results in:

8a $$\begin{aligned}&c^{2}=\frac{eE_1 }{2\rho r} \end{aligned}$$

8b $$\begin{aligned}&\frac{\partial ^{2}p}{\partial t^{2}}+\frac{1}{\tau }\cdot \frac{\partial p}{\partial t}=c^{2}\cdot \frac{\partial ^{2}p}{\partial x^{2}} \end{aligned}$$

This is a wave equation with a linear dissipation term. When $\eta$ tends to infinity, $\tau$ does too and Eq. (8b) becomes the well-known equation for (undamped) pressure pulses inside elastic pipes, like in a water hammer.

When the initial and boundary conditions are convenient, Eq. (8b) can be solved by separation of variables and Sturm–Liouville series. For the conditions set in (9), this becomes an eigenvalue problem that allows calculating the decaying rate of the pressure wave. These eigenvalues define the inherent damping ability of the material.

9 $$\begin{aligned} \left\{ {{\begin{array}{l} {p\left( {0,t} \right) =0} \\ {p\left( {L,t} \right) =0} \\ {p\left( {x,0} \right) =f\left( x \right) } \\ {\frac{\partial p}{\partial t}\left( {x,0} \right) =g\left( x \right) } \\ \end{array} }} \right. \end{aligned}$$

The eigenvalues, which are the spatial frequencies, are: $\lambda _n =\left( {\frac{n\pi }{L}} \right) ^{2}, n\in {\mathbb {N}}$, and the formal solution to (8) is:

10 $$\begin{aligned} p\left( {x,t} \right)= & {} \sum \nolimits _{n=1}^\infty ( C_{1n} \exp \left( {\mu _{1n} t} \right) \nonumber \\&+\,C_{2n} \exp \left( {\mu _{2n} t} ) \right) \sin \frac{n\pi x}{L} \end{aligned}$$

where $\mu _{1n,2n}=\frac{1}{2\tau }\pm \sqrt{\Delta _{\mathrm{n}}}$ with the discriminant

11 $$\begin{aligned} {\Delta }_\mathrm{n} =\frac{1}{4\tau ^{2}}- \lambda _n c^{2}= \frac{E_1^2 }{4\eta ^{2}}-\frac{n^{2}\pi ^{2}}{L^{2}}\cdot \frac{eE_1 }{2\rho r} \end{aligned}$$

Note that $\mu _{1n,2n}$ are always negative. Hence, if the discriminant ${\Delta }_\mathrm{n}$ is negative, the corresponding term is underdamped. If the discriminant is zero, the corresponding term is critically damped. If the discriminant is positive, the corresponding term is overdamped. Note that the sequence of the values of this discriminant (11) is decreasing and unbounded. In consequence, there will be infinite negative terms (underdamped). However, for low values of *n*,  the discriminant could be positive and hence the lowest spatial frequencies may be overdamped. This is when

12 $$\begin{aligned} \tau ^{2}<\frac{L^{2}}{n^{2}\pi ^{2}}\cdot \frac{\rho r}{2eE_1 } \end{aligned}$$

For underdamped cases, the solution to (8) can be written as a function of time and space:

13a $$\begin{aligned} p\left( {x,t} \right)= & {} e^{-\frac{t}{2\tau }}\cdot \phi \left( {x,t} \right) \end{aligned}$$

13b $$\begin{aligned} p\left( {x,t} \right)= & {} e^{-\frac{x_t }{2\tau \cdot c}}\cdot \phi \left( {x,t} \right) \end{aligned}$$

where $x_t =c\cdot t$ is the position of the pressure pulse and $x\in \left[ {0,L} \right]$ is the spatial coordinate.

If $\omega _n =\sqrt{-{\Delta }_\mathrm{n} }, C_n$ and $\delta _n$ are constants to be determined from the initial conditions, the auxiliary function $\phi \left( {x,t} \right)$ is defined as follows:

13c $$\begin{aligned} \phi \left( {x,t} \right) =\sum \nolimits _{n=1}^\infty C_n \cos \left( {\omega _n t+\delta _n } \right) \sin \frac{n\pi x}{L} \end{aligned}$$

$\phi \left( {x,t} \right)$ is an undamped wave, and most importantly a periodic function in both time and space. Consequently, it does not show any irreversible decay, and therefore, the decay rate of the wave is the negative exponential in (13a) with constant of time $2{\tau }$.

For those cases where condition (12) is not fulfilled, a number of overdamped terms arise. This happens for values of *n* lower than a critical value *k*, which is $L/\left( {2c\pi \tau } \right)$. Then, the solution becomes:

14 $$\begin{aligned} p\left( {x,t} \right)= & {} \sum \nolimits _{n=1}^k \left[ {A_n e^{\mu _{1n} t}+B_n e^{\mu _{2n} t}} \right] \nonumber \\&+\,e^{-\frac{t}{2\tau }} \mathop \sum \nolimits _{n=k+1}^\infty C_n \cos ( \omega _n t +\delta _n ) \sin \frac{n\pi x}{L} \end{aligned}$$

Note that $\mu _{2n}<-1/2\tau<\mu _{1n} <0$, so the wave will show an overdamped part and an underdamped part. One part of the overdamped terms decay faster than the undamped terms, and the other part decays slower. The dominant decay rate of the overdamped part is controlled by $\mu _{11} >-1/2\tau$.

### Pressure propagation using the Zener model

Time derivation of the Zener model (2) gives:

15 $$\begin{aligned} \frac{\partial ^{2}\sigma }{\partial t^{2}}+\frac{1}{\tau }\cdot \frac{\partial \sigma }{\partial t}=(E_0 +E_1 ) \frac{\partial ^{2}\varepsilon }{\partial t^{2}}+\frac{E_0 }{\tau }\cdot \frac{\partial \varepsilon }{\partial t} \end{aligned}$$

The continuity Eq. (4) gives:

16 $$\begin{aligned} \frac{\partial u}{\partial x}=-2\cdot \frac{\partial \varepsilon }{\partial t} \end{aligned}$$

And, after time derivation:

17 $$\begin{aligned} \frac{\partial ^{2}u}{\partial t\partial x}=-2\cdot \frac{\partial ^{2}\varepsilon }{\partial t^{2}} \end{aligned}$$

Combining (15) with (16, 17), one has:

18 $$\begin{aligned} (E_0 +E_1 ) \frac{\partial ^{2}u}{\partial t\partial x}+\frac{E_0 }{\tau }\cdot \frac{\partial u}{\partial x}=-2\cdot \frac{\partial ^{2}\sigma }{\partial t^{2}}-\frac{2}{\tau }\cdot \frac{\partial \sigma }{\partial t} \end{aligned}$$

And, deriving respect to time once again:

19 $$\begin{aligned} (E_0 +E_1 ) \frac{\partial ^{3}u}{\partial t^{2}\partial x}+\frac{E_0 }{\tau }\cdot \frac{\partial ^{2}u}{\partial t\partial x}=-2\cdot \frac{\partial ^{3}\sigma }{\partial t^{3}}-\frac{2}{\tau }\cdot \frac{\partial ^{2}\sigma }{\partial t^{2}} \end{aligned}$$

The balance of the momentum, assuming no convective acceleration and no friction (5) and deriving respect to space gives:

20 $$\begin{aligned} \rho \frac{\partial ^{2}u}{\partial t\partial x}=-\frac{\partial ^{2}p}{\partial x^{2}} \end{aligned}$$

And deriving again respect to time:

21 $$\begin{aligned} \rho \frac{\partial ^{3}u}{\partial t^{2}\partial x}=-\frac{\partial ^{3}p}{\partial t\partial x^{2}} \end{aligned}$$

If $c_0^2 =\frac{E_0 e}{2\rho r}$ and $c_1^2 =\frac{E_1 e}{2\rho r}$, one can now combine Eq. (19) with (3, 20 and 21):

22a $$\begin{aligned}&c=\sqrt{c_0^2 +c_1^2 } \end{aligned}$$

22b $$\begin{aligned}&c^{2}\cdot \frac{\partial ^{3}p}{\partial t\partial x^{2}}+\frac{c_0^2 }{\tau }\cdot \frac{\partial ^{2}p}{\partial x^{2}}=\frac{\partial ^{3}p}{\partial t^{3}}+\frac{1}{\tau }\cdot \frac{\partial ^{2}p}{\partial t^{2}} \end{aligned}$$

This third-order partial differential equation appears as a linearized model in nonlinear acoustics, under the name of Moore–Gibson–Thompson equation. Taking the dominant terms (those of third order), this is a wave equation with a wave velocity *c*. We propose the next formal solution, which is analogous to the one found in (13):

23 $$\begin{aligned} p\left( {x,t} \right) =\sum \nolimits _{n=1}^\infty e^{-\frac{t}{\tau _n }}\cdot \psi _n \left( x \right) \end{aligned}$$

where $\psi$ is the solution of the equation:

24 $$\begin{aligned} \psi _n^{{\prime }{\prime }} \left( x \right) +\lambda _n \cdot \psi _n \left( x \right) =0 \end{aligned}$$

and

25 $$\begin{aligned} \lambda _n =\frac{1}{\tau _n^2 }\cdot \frac{1-\frac{\tau }{\tau _n }}{\frac{\tau }{\tau _n }\cdot (c_0^2 +c_1^2 )-c_0^2 } \end{aligned}$$

When pressure is zero, (25) gives $\lambda _n =\left( {\frac{n\pi }{L}} \right) ^{2}$ and the function $\psi$ is:

26 $$\begin{aligned} \psi _n \left( x \right) =C_n \cdot \sin \frac{n\pi x}{L} , n\in {\mathbb {N}} \end{aligned}$$

where *L* is the length of the spatial domain and $$C_n $$ every constant. From (25) one gets the cubic characteristic equation, for the unknown $$1/\tau _n $$:

27 $$\begin{aligned} \frac{1}{\tau _n^3 }-\frac{1}{\tau }\cdot \frac{1}{\tau _n^2 }+\lambda _n \left( {c_0^2 +c_1^2 } \right) \cdot \frac{1}{\tau _n }-\frac{1}{\tau }\cdot \lambda _n c_0^2 =0 \end{aligned}$$

This polynomial does not have any negative real root. Using the formulae of Cardano, the roots of this polynomial are:

28a $$\begin{aligned} \frac{1}{\tau _{n,1}}= & {} \frac{1}{3\tau }+S_1 +S_2 \end{aligned}$$

28b $$\begin{aligned} \frac{1}{\tau _{n,2}}= & {} \frac{1}{3\tau }-\frac{S_1 +S_2 }{2}+\frac{\sqrt{3}}{2}i\left( {S_2 -S_1 } \right) \end{aligned}$$

28c $$\begin{aligned} \frac{1}{\tau _{n,3}}= & {} \frac{1}{3\tau }-\frac{S_1 +S_2 }{2}-\frac{\sqrt{3}}{2}i\left( {S_2 -S_1 } \right) \end{aligned}$$

where

29a $$\begin{aligned} S_1= & {} \frac{1}{3\tau }\root 3 \of {R+\sqrt{R^{2}+Q^{3}}}\hbox { };\hbox { }\nonumber \\ S_2= & {} \frac{1}{3\tau }\root 3 \of {R-\sqrt{R^{2}+Q^{3}}} \end{aligned}$$

29b $$\begin{aligned} R= & {} \frac{9}{2}\cdot {\tau }^{2}\lambda _n \left( {2c_0^2 -c_1^2 } \right) +1 \end{aligned}$$

29c $$\begin{aligned} Q= & {} 3{\tau }^{2}\lambda _n \left( {c_0^2 +c_1^2 } \right) -1 \end{aligned}$$

The lowest value of the real part of $1/\tau _n$ controls the decaying rate and that is:

30 $$\begin{aligned} \frac{1}{\tau _n }=\hbox {min}\left( {\frac{1}{3\tau }+S_1 +S_2 , \frac{1}{3\tau }-\frac{S_1 +S_2 }{2}} \right) \end{aligned}$$

The value of $$S_1 +S_2 $$ decreases as *n* increases and tends to a constant value depending on the parameters of the material model:

31 $$\begin{aligned} \mathop {\lim }\nolimits _{n\rightarrow \infty } \left( {S_1 +S_2 } \right) =\frac{1}{3\tau }\cdot \frac{2c_0^2 -c_1^2 }{c_0^2 +c_1^2 }=\frac{1}{3\tau }\cdot \left( {2-\frac{3{E}_1 }{{E}_0 +{E}_1 }} \right) \end{aligned}$$

Therefore, the limiting value of the constant $1/\tau _n$can be stated from (30) and (31):

32 $$\begin{aligned} \frac{1}{\tau _{n\rightarrow \infty } }=\hbox {min}\left( {\frac{1}{\tau }\cdot \frac{{E}_0 }{{E}_0 +{E}_1 } , \frac{1}{2\tau }\cdot \frac{{E}_1 }{{E}_0 +{E}_1 }} \right) \end{aligned}$$

This limiting value is known as the “essential spectrum” of the problem. Observe in the expression (30) that, for a range of cases, the value of $\tau _n$ is almost independent on *n* and the exponential part of the solution (23) can be taken out of the sum. This limiting value of $1/\tau _n$ can be approximated by (32), and this is when:

33 $$\begin{aligned} \tau ^{2}\cdot c^{2}\gg 1\hbox { m}^{2} \end{aligned}$$

The same is true when the pulse width is very short compared with the length of the space considered, because the lowest harmonic terms in (23) are very weak.

Note that the imaginary part of (28b,28c) represents the harmonics of an oscillation with increasing frequency. Therefore, the solution for (23) can be written as a function of time and space:

34a $$\begin{aligned} p\left( {x,t} \right)= & {} e^{-\frac{t}{\tau _{eq} }}\cdot \xi \left( {x,t} \right) \end{aligned}$$

34b $$\begin{aligned} p\left( {x,t} \right)= & {} e^{-\frac{x_t }{c\cdot \tau _{eq} }}\cdot \xi \left( {x,t} \right) \end{aligned}$$

where $x_t =c\cdot t$ is the position of the pressure pulse and $x\in \left[ {0,L} \right]$ is the spatial coordinate. $\xi \left( {x,t} \right)$ is a bounded function which is periodic in both time and space or damped in time and periodic in space, and $\tau _{eq}$ can be calculated by (35).

35 $$\begin{aligned} \tau _{eq} =\hbox {max}\left( {\tau \cdot \frac{E_0 +E_1 }{E_0 } , 2\tau \cdot \frac{E_0 +E_1 }{E_1 }} \right) \end{aligned}$$

When condition (33) is not fulfilled, the value of $\tau _n$ in Eq. (30) depends on the value of *n*. In that case, the exponential part of the solution (23) can’t be taken out of the sum and the decaying rate becomes a weighted sum of *n* exponential decays limited by $n=1$ (lowest decaying rate) and $n\rightarrow \infty$ (highest decaying rate).

In summary, expressions (13) and (34-35) give the decaying rate of a pressure wave along a tube when condition (33) is fulfilled only for the Zener model. Note that both expressions refer to the pressure, which is proportional to stress $\sigma$ (3); therefore, the decaying rate for the stress is the same for pressure and stress. When (33) is not fulfilled, the decaying rate is bounded between the decaying rate calculated for $n=1$ and the one calculated for $n\rightarrow \infty$ with Eq. (30).

### Numerical simulations

The formulae previously developed are compared to numerical one-dimensional simulations. We propose the problem with a realistic blood pressure pulse arising from a heartbeat. The pressure wave propagates along a straight viscoelastic pipe of length L (Fig. 1d), with pulsatile inlet *f*(*t*) as shown in Eq. (36):

36 $$\begin{aligned} f\left( t \right) =\left\{ {{\begin{array}{ll} \frac{p_\mathrm{max} }{2}\left( {1-\hbox {cos}\left( {\frac{2 \pi }{T} t} \right) } \right) &{}, t\le T \\ 0&{}, t>T \\ \end{array} }} \right. \end{aligned}$$

In consequence, for the Maxwell material model, the set of conditions of the problem are defined in (37) as:

37 $$\begin{aligned} \left\{ {{\begin{array}{l} {{\begin{array}{l} {\frac{\partial ^{2}p}{\partial t^{2}}+\frac{1}{\tau }\cdot \frac{\partial p}{\partial t}=c^{2}\cdot \frac{\partial ^{2}p}{\partial x^{2}}} \\ {p\left( {0,t} \right) =f\left( t \right) }\\ \end{array} }} \\ {p\left( {L,t} \right) =0}\\ {p\left( {x,0} \right) =0}\\ {\frac{\partial p}{\partial t}\left( {x,0} \right) =0}\\ \end{array} }} \right. \end{aligned}$$

The partial differential equation (PDE) is second order both in time and in space. It has been solved by the method of lines (Saucez et al. 2004). The space dimension has been discretized using finite differences, by centered formulas of second order. This gives a system of ordinary differential equation (ODE) in time, where *h* is the space step of the discretization.

38 $$\begin{aligned} \frac{d^{2}p_i }{\mathrm{d}t^{2}}+\frac{1}{\tau }\frac{\mathrm{d}p_i }{\mathrm{d}t}=c^{2}\left( {\frac{p_{i+1} -2p_i +p_{i-1} }{h^{2}}} \right) , i=1\ldots N \end{aligned}$$

To reduce these second-order ODEs to first-order ones, one takes:

39 $$\begin{aligned} \frac{\mathrm{d}p_i }{\mathrm{d}t}\left( t \right) =r_i \left( t \right) \end{aligned}$$

This gives 2N equations:

40 $$\begin{aligned} \left\{ {{\begin{array}{l} {\frac{\mathrm{d}p_i }{\mathrm{d}t}=r_i } \\ {\frac{dr_i }{\mathrm{d}t}=c^{2}\left( {\frac{p_{i+1} -2p_i +p_{i-1} }{h^{2}}} \right) -\frac{1}{\tau }r_i } \\ \end{array} }} \right. , i=1\ldots N \end{aligned}$$

And the conditions:

41 $$\begin{aligned} p_0 =f\left( t \right) ; p_{N+1} =0 \end{aligned}$$

The ODE system (40,41) is solved using the Dormand–Prince method (Dormand and Prince 1980), programmed in MATLAB (ode45).

For the Zener model, the set of conditions of the problem are defined in (42), analogous to (37), with the pressure pulse defined previously in (36):

42 $$\begin{aligned} \left\{ {\begin{array}{l} \left( {c_0^2 +c_1^2 } \right) \frac{\partial ^{3}p}{\partial t\partial x^{2}}+\frac{c_0^2 }{\tau }\cdot \frac{\partial ^{2}p}{\partial x^{2}}=\frac{\partial ^{3}p}{\partial t^{3}}+\frac{1}{\tau }\cdot \frac{\partial ^{2}p}{\partial t^{2}} \\ p\left( {0,t} \right) =f\left( t \right) \\ p\left( {L,t} \right) =0 \\ \frac{\partial ^{2}p}{\partial t\partial x}\left( {L,0} \right) =0 \\ p\left( {x,0} \right) =0 \\ \frac{\partial p}{\partial t}\left( {x,0} \right) =0 \\ \frac{\partial ^{2}p}{\partial t^{2}}\left( {x,0} \right) =0 \\ \end{array}} \right. \end{aligned}$$

Taking into account (39) and (42):

43 $$\begin{aligned} \frac{\partial ^{2}p_i }{\partial t ^{2}}\left( t \right) =\frac{\partial r_i }{\partial t}\left( t \right) =s_i \left( t \right) \end{aligned}$$

The PDE gives the system of 3*N* ODE ($i = 1{\ldots }\hbox {N}$):

44 $$\begin{aligned} \left\{ \begin{array}{ll} {\frac{\mathrm{d}p_i }{\mathrm{d}t}=r_i }&{} {i=1\ldots N} \\ {\frac{\mathrm{d}r_i }{\mathrm{d}t}=s_i }&{} { } \\ \frac{\mathrm{d}s_i }{\mathrm{d}t}=\frac{c_0^2 }{\tau \left( {h} \right) ^{2}}\cdot \left( {p_{i+1} -2p_i +p_{i-1} } \right) \\ \qquad \quad +\,\frac{c_0^2 +c_1^2 }{\left( {h} \right) ^{2}}\cdot \left( {r_{i+1} -2r_i +r_{i-1} } \right) -\frac{1}{\tau }\cdot s_i &{} \\ \end{array} \right. \end{aligned}$$

And the boundary conditions give:

45 $$\begin{aligned} p_0 =f\left( t \right) ; p_{N+1} =0 ;r_0 ={f}'(t); r_{N+1}=r_{N-1} \end{aligned}$$

The ODE system (44,45) is solved using the same MATLAB code.

### Mechanical testing and model adjustment

The ability to damp pressure waves was tested in polydimethylsiloxane (PDMS, Dow Corning, Midland, MI), a common material used in medical applications. Maxwell and Zener models were adjusted to represent the viscoelastic behavior of PDMS using a stress/strain experiment. Stress/strain tests were carried out using Instron$^{{\textregistered }}$ ElectroPulsTM E3000a tensile rig. Force was obtained from load cells and displacement obtained from grip displacement. Briefly, a uniaxial sinusoidal displacement at 1Hz was applied to a flattened half cylinder of PDMS of 14.28 mm diameter and 2.37 mm thickness. This tube was cut into slices of 10 mm width and straightened to a length of 37.38 mm. The PDMS sample was held to the uniaxial test machine allowing a length between fixations of 20 mm. (See supplemental Video 1) Tube flattening provoked a 20% compression on the exterior face and a 20% tension on the interior face. The displacement applied deformed the sample up to 7% to obtain stress/time and strain/time curves. Our experiment measured the average tensile strength along the tube. We performed a series of simulations to confirm that the significant thickness of our pipe (0.166 thickness-to-diameter ratio) would not affect our results. Indeed, we observed that, for ratios between 0.1 and 0.2, the error between theoretical and average stress ranged approximately $\pm \,3\%$.

The stress/strain experiment was simulated in parallel to estimate the material models’ parameters that fit best the experimental data. These simulations were run using the Dormand–Prince method, with the experimental strain/time curve as an input and the stress/time curves as an output. The error between the experimental and the simulated stress/time curves was calculated as the average of the orthogonal distance from each point of the experimental data to the simulated curve. This error was minimized using the Nelder–Mead simplex method (Lagarias et al. 1998) to find the material models’ parameters that best represented the material behavior.

## Results

### Formulae validation

This first section is devoted to test and discuss the validity of the models and the formulae developed in the methods section. These calculations only prove the validity of the mathematical model, and do not represent any specific physical condition. That is, these simulations calculate the pressure wave damping for certain values of $\tau$, *c*, *T* and *L* without aiming at any particular material.

**Fig. 2:**
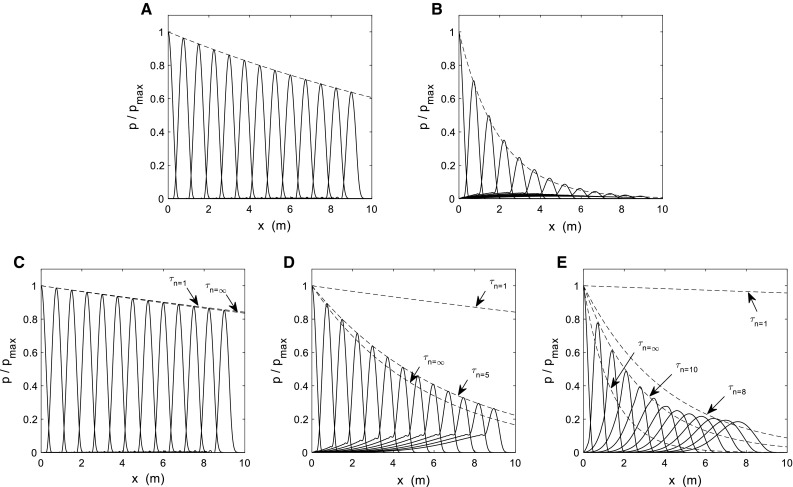
**a** Decaying wave for the Maxwell material model. Solid lines are the pressure along the pipe, for increasing times (every 0.15 s). The dashed line is (13b). Values: $\tau$ = 2 s, *c* = 5 m/s, *T* = 0.2 s and *L* = 10 m. **b** Decaying wave for the Maxwell material model. Solid lines are the pressure along the pipe, for increasing times (every 0.15 s). The dashed line is (13b). Values: $\tau$ = 0.2 s, *c* = 5 m/s, *T* = 0.2 s and *L* = 10 m. **c** Decaying wave for the Zener material model. Solid lines are the pressure along the pipe, for increasing times (every 0.15 s). Dashed lines are the exponential decaying for $n=1$ and for $n\rightarrow \infty$ calculated with (30) and (32), respectively. Values: $\tau$ = 2 s, $c_{0}$ = 4 m/s, $c_{1}$ =3 m/s, *T* = 0.2 s and *L* = 10 m. **d** Decaying wave for the Zener material model: Solid lines are the pressure along the pipe, for increasing times (every 0.15 s). Dashed lines are the exponential decaying for $n=1$ and $n=5$ calculated with (30) and for $n\rightarrow \infty$ calculated with (32). Values: $\tau$ = 0.2 s, $c_{0}$ = 4 m/s, $c_{1}$ =3 m/s, *T* = 0.2 s and *L* = 10 m. **e** Decaying wave for the Zener material model: Solid lines are the pressure along the pipe, for increasing times (every 0.15 s). Dashed lines are the exponential decaying for $n=1$, $n=8$ and $n=10$ calculated with (30) and for $n\rightarrow \infty$ calculated with (32). Values: $\tau$ = 0.05 s, $c_{0}$ = 4 m/s, $c_{1}$ =3 m/s, *T* = 0.2 s and *L* = 10 m

Figure 2a shows the result of a simulation using the Maxwell material model, for a set of values where all frequencies are underdamped. In this case, $\tau$ = 2 s, *c* = 5 m/s, *T* = 0.2 s and *L* = 10 m. The dashed line shows the exponential decay along the spatial axis, following Eq. (13b). Solid lines represent the pressure pulse along the pipe in increments of 0.15 s in time. The displacement of the pulse has been simulated before it reaches the end of the pipe, to avoid end-of-pipe reflection effects. The exponential decay adjusts perfectly to the pressure wave damping.

Figure 2b shows the result of a simulation using the Maxwell material model, for a set of values where some frequencies are overdamped. In this case, $\tau$ = 0.2 s, *c* = 5 m/s, *T* = 0.2 s and *L* = 10 m. The overdamped terms provoke that the pressure profiles exceed the exponential. In the solution shown in Fig. 2b, the smooth wave that develops at the bottom corresponds to the slow-decaying terms: For these overdamped terms, the dominant term (14) is $A_1 \cdot \exp \left( {\mu _{11} \cdot t} \right)$, with $\mu _{11} =-\,0.555\hbox { s}^{-1}$. The higher-frequency terms (the sharp peak of the pressure pulse) disappear faster than the low-frequency terms (the smooth wave remaining). The exponential decay adjusts fairly to the pressure wave damping.

Figure 2c shows the result of a simulation using the Zener material model, for a case where ${E}_{1} < 2\cdot { E}_{0}$. In this case: $\tau = 2\hbox { s}$, $\hbox {c}_{0} = 4\hbox { m/s}$, $\hbox {c}_{1} = 3\hbox { m/s}$,, $\hbox {T} = 0.2\hbox { s}$ and $\hbox {L} = 10\hbox { m}$. According to (22a), $\hbox {c} = 5\hbox { m/s}$, which is the same value that is used in Fig. 2a, b. Solid lines represent the pressure pulse along the pipe in increments of 0.15 s in time. Dashed lines show the exponential decay along the spatial axis, for $n=1$ and for $n\rightarrow \infty$ calculated with (30) and (32), respectively. The exponential decay is almost independent of *n* because condition (33) is fulfilled. The limiting value (known as essential spectrum and with highest decaying rate) is $\tau _\infty =11.11$ s, only slightly lower than $\tau _1 =11.59$ s (lowest decaying rate). In this case then, the decaying rate can be calculated with Eq. (34b), and therefore, the essential spectrum given by (35) is a good predictor for the decaying rate.

Figure 2d shows the result of another simulation using the Zener material model, for a case where $\tau = 0.2\hbox { s}$, $\hbox {c}_{0} = 4\hbox { m/s}$, $\hbox {c}_{1} = 3\hbox { m/s}$, $\hbox {T} = 0.2\hbox { s}$ and $\hbox {L} = 10\hbox { m}$. In this case, condition (33) is not fulfilled as $\tau ^{2}\cdot c^{2}=1\hbox { m}^{2}$. The dashed lines show the exponential decay for different values of $\tau _n$: the extreme values ($n=1$ and $n\rightarrow \infty$) and an intermediate $\tau _n$ for $n=5$, the value of n that best adjusts to the true decaying rate of the pressure wave . The maximum value of $\tau _n$ was calculated using (30) for $n=1$ and is 11.606 s. The intermediate value was calculated also using (30) for $n=5$ and is 1.332 s. The minimum, for $n\rightarrow \infty$, is 1.111 s, calculated by (32). This last value does not fit properly the decaying rate but could be used as a first approximation for the sharpest part of the wave. The exponential decay calculated for $n=5$ does fit very well the wave decaying rate but it is a particular case for these specific conditions; varying the material model parameters or the geometry would change the value of *n* that best fits the wave decaying rate. In conclusion, when condition (33) is mildly not fulfilled, the spectrum given by (35) for $n\rightarrow \infty$ approximates fairly to the true decaying rate.

When condition (33) is definitely not fulfilled, none of the previously described estimates work. In Fig. 2e, we show a case where $\tau = 0.05\hbox { s}$, $\hbox {c}_{0} = 4\hbox { m/s}$, $\hbox {c}_{1} = 3\hbox { m/s}$, and $\hbox {T} = 0.2\hbox { s}$, hence $\tau ^{2}\cdot c^{2}=0.0625\hbox { m}^{2}$ and condition (33) is not satisfied. The figure shows the exponential decay for the extreme values of $\tau _n$ and two intermediate values for $n=8$ and for $n=10$, which are calculated and represented analogously to those shown in Fig. 2d. For $n=1$, the value of $\tau _n$ is 45.11 s, for $n=8$ and for $n=10$ the values of $\tau _n$ are 0.820 s and 0.588 s, respectively, and the for $n\rightarrow \infty$, the value is 0.278 s. The figure shows how neither the maximum nor the minimum values of $\tau _n$ are useful for predicting the pulse attenuation as none of the estimated decays adjusts to the true decaying rate of the wave. Also, none of the intermediate values completely fit the data as the true decaying rate is the result of a weighted sum of *n* exponential decays instead of a single exponential decay. The method finds its true limit when $\tau ^{2}\cdot c^{2}<1\hbox { m}^{2}$.

### Pulse wave attenuation prediction

Formulae (13–14 and 34–35) give an exponential pattern of the decay rate of a pulse for the Maxwell and the Zener material models. This can be written, as a function of the space:

46 $$\begin{aligned} p\left( x \right) =p_0 \cdot \exp \frac{-x}{c\cdot \tau _{eq} } \end{aligned}$$

where *c* is the velocity of the wave, this is (8a) for the Maxwell model and (22a) for the Zener model.

$\tau _{\mathrm{eq}}$ is, for the Maxwell model:

47 $$\begin{aligned} \tau _{\hbox {eq}} =\left\{ {{\begin{array}{ll} 2\tau ,&{}\quad 4\tau ^{2}\pi ^{2}\cdot \frac{c^{2}}{L^{2}}\ge 1 \\ \frac{2\tau }{1-\sqrt{1-4\tau ^{2}\pi ^{2}\cdot \frac{c^{2}}{L^{2}}}}>2\tau ,&{}\quad 4\tau ^{2}\pi ^{2}\cdot \frac{c^{2}}{L^{2}}<1 \\ \end{array} }} \right. \end{aligned}$$

And, for the Zener model if $\tau ^{2}\cdot c^{2}\gg 1\hbox {m}^{2}$:

48 $$\begin{aligned} \tau _{\mathrm{eq}} \approx \left\{ {{\begin{array}{ll} 2\tau \cdot \frac{E_0 +E_1 }{E_1 }\in \left( {2\tau ,3\tau } \right) ,&{}E_1 <2E_0 \\ \tau \cdot \frac{E_0 +E_1 }{E_0 }\ge 3\tau ,&{}E_1 \ge 2E_0 \\ \end{array} }} \right. \end{aligned}$$

Note that, for this last model, the time constant is only valid for the steepest part of the pulse.

When the interest is to study the pulse attenuation, the length is a critical limitation. The desired attenuation *a* is defined as the pressure damping between inlet ($\hbox {x}=0$, p0) and outlet ($\hbox {x}=\hbox {L}$, p(L)):

49 $$\begin{aligned} a=1-\frac{p_{OUT} }{p_{IN} }=1-\frac{p\left( L \right) }{p_0 } \end{aligned}$$

Combining (46) and (49), one can obtain the analytical expression of the length *L* of material required for a certain attenuation *a*.

50 $$\begin{aligned} L=c\cdot \tau _{eq} \cdot \ln \frac{1}{1-a} \end{aligned}$$

### Experimental determination of the material models’ parameters

**Fig. 3:**
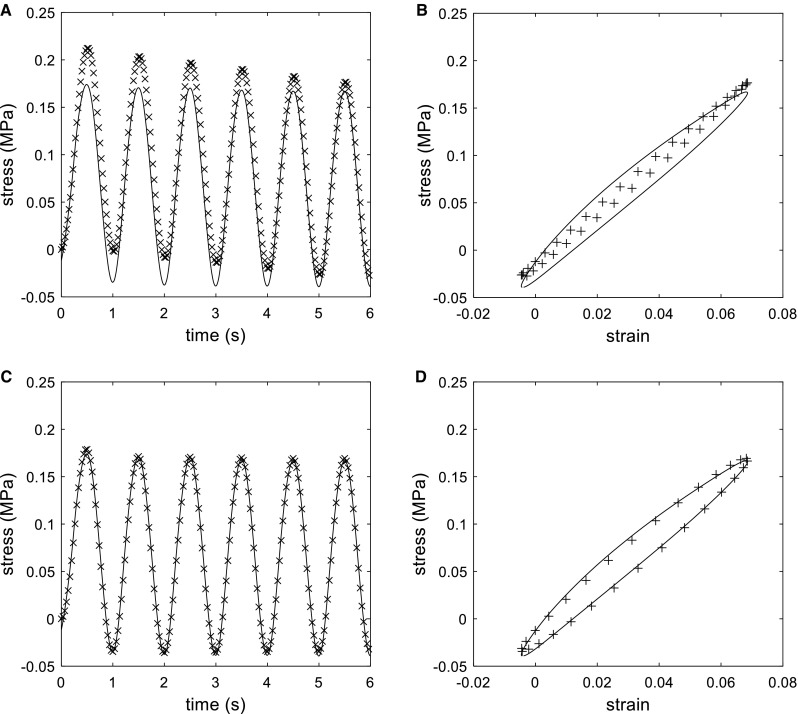
**a** Best adjustment found for Maxwell model. Comparison of stress/time curves between experimental data (solid line) and simulation (crosses) with ${E}_{1} = 2.827\hbox { MPa}$ and $\tau = 13.38\hbox { s}$. **b** Best adjustment found for Maxwell model. Comparison of hysteresis in stress/strain curves for $5<{t}<6\hbox {s}$ between experimental data (solid line) and simulation (crosses) with ${E}_{1} = 2.827\hbox { MPa}$ and $\tau = 13.38\hbox { s}$. **c** Best adjustment found for Zener model. Comparison of stress/time curves between experimental data (solid line) and simulation (crosses) with ${E}_{1} = 0.9365\hbox { MPa}$, ${E}_{0} = 2.114\hbox { MPa}$ and $\tau = 0.2611\hbox { s}$. **d** Best adjustment found for Zener model. Comparison of hysteresis in stress/strain curves for $5<{t}<6\hbox {s}$ between experimental data (solid line) and simulation (crosses) with ${E}_{1} = 0.9365\hbox { MPa}$, ${E}_{0} = 2.114\hbox { MPa}$ and $\tau = 0.2611\hbox { s}$

Maxwell and Zener models were used to characterize the mechanical behavior of our PDMS samples. As seen in Fig. 3a, b, the best fit for the Maxwell poorly adjusted to the experimental stress/strain and stress/time cycles. The best adjustment found for the Maxwell model was ${E}_{1} = 2.827\hbox { MPa}$ and $\tau = 13.38\hbox { s}$. In the first cycle there was an offset of approximately +0.025 MPa in the lower side of the cycle and of +0.05 MPa in the higher side of the cycle, and although these differences decreased in the next cycles, the tendency of the simulation did not fit the experimental curve. On the other hand, the Zener model was able to properly adjust the stress/strain and stress/time cycles of the PDMS sample. This is shown in Fig. 3c, d. In this case, the best fit was for the parameters ${E}_{1}=0.9365\hbox { MPa}$, ${E}_{0} = 2.114\hbox { MPa}$ and $\tau = 0.2611\hbox { s}$. The rest of the work was done using the Zener model and the parameters obtained.

### Numerical simulations of pressure wave damping by PDMS

Simulations were performed to estimate how a straight PDMS prosthesis would be able to damp a pressure pulse. The prosthesis was a straight cylinder of 4.0 mm in radius and 0.5 mm in thickness. The wave velocity *c*, calculated with (22a), was 13.808 m/s, and the fluid density was set at $1000\hbox { kg/m}^{3}$. This simulation would correspond to a cylindrical PDMS device implanted in a straight vessel such as the common carotid artery, which has an approximate radius of 4.0 mm. The period of the pulse was set at 0.2 s, which is the amplitude of a systole, and the simulated length was enough for the wave to run the entire cylinder. The length of the simulation was calculated as the multiplication of the pulse period and the propagation speed *c*. The results showed that our PDMS prosthesis would only be able to damp around 4% in the first meter of device. This is consistent with the attenuation predicted by formulae (46,48), which matches with the results of the numerical simulation.

**Fig. 4:**
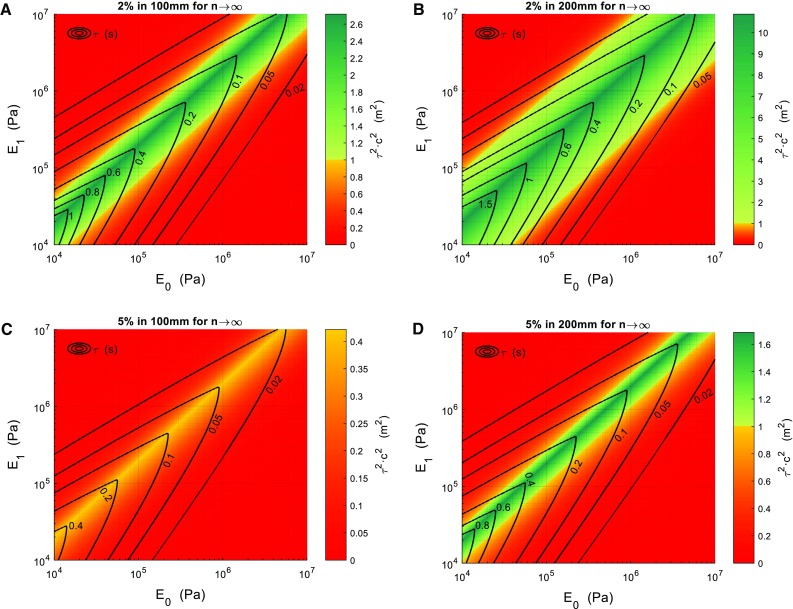
**a** Surface that correlates the values of ${E}_{0}$ (x axis), ${E}_{1}$ (y axis) and $\tau$ (contour lines) to obtain a 2% pressure attenuation in a 100-mm-long pipe. Contour lines represent the values for $\tau$, and the color map represents the value of $\tau ^{2}\cdot c^{2}$ to check for condition (33) fulfillment. **b** Surface that correlates the values of ${E}_{0}$ (x axis), ${E}_{1}$ (y axis) and $\tau$ (contour lines) to obtain a 2% pressure attenuation in a 200-mm-long pipe. Contour lines represent the values for $\tau$, and the color map represents the value of $\tau ^{2}\cdot c^{2}$ to check for condition (33) fulfillment. **c** Surface that correlates the values of ${E}_{0}$ (x axis), ${E}_{1}$ (y axis) and $\tau$ (contour lines) to obtain a 5% pressure attenuation in a 100-mm-long pipe. Contour lines represent the values for $\tau$, and the color map represents the value of $\tau ^{2}\cdot c^{2}$ to check for condition (33) fulfillment. **d** Surface that correlates the values of ${E}_{0}$ (x axis), ${E}_{1}$ (y axis) and $\tau$ (contour lines) to obtain a 5% pressure attenuation in a 200-mm-long pipe. Contour lines represent the values for $\tau$, and the color map represents the value of $\tau ^{2}\cdot c^{2}$ to check for condition (33) fulfillment

Finally, Fig. 4 shows four combinations of the Zener model parameters required to obtain different pressure attenuations (2–5%) in a 4.0 mm in radius, 0.5 mm in thickness and 100- to 200-mm-long cylinder. This was not done using the Maxwell material model since results depicted in Fig. 3A and B showed that the Maxwell model did not fit properly to our requirements. Values of ${E}_{0}$ and ${E}_{1 }$ were set between 0.01 and 10MPa and values of $\tau$ were calculated so that Eqs. (46,48) fitted the proposed pressure damping. The value of $\tau ^{2}\cdot c^{2}$ was also computed to check for condition (33) fulfillment. In those areas where $\tau ^{2}\cdot c^{2}<1\hbox { m}^{2}$, the proposed attenuation cannot be accomplished with any combination of the material model parameters (${E}_{0}$, ${E}_{1}$ and $\tau$) in the ranges represented in the figure. As seen in Fig. 4a, there is a narrow range of material properties that allow for a 2% attenuation in a 100-mm-long cylinder. This attenuation is easier to reach when the cylinder is expanded up to 200 mm (Fig. 4b). As seen in Fig. 4c, the condition $\tau ^{2}\cdot c^{2}\ge 1\hbox { m}^{2}$ is never satisfied and hence an attenuation of 5% in 100 mm is impossible within the range of ${E}_{0}$, ${E}_{1}$ and $\tau$ that we have studied. There is, however, a narrow range of material properties in which a 5% damping is possible if the cylinder is extended to 200 mm (Fig. 4d).

## Discussion

Aortic stiffness, increased pulse wave velocity, and aging are intrinsically connected (O’Rourke 1990). Changes in blood flow profiles can affect the vessel’s functionality and hence the organ that this vessel feeds (Balcells et al. 2005). These changes, which have been classically described in the most relevant vessels for cardiovascular medicine (Li et al. 2005), i.e., coronary arteries (Chatzizisis et al. 2007; Dancu and Tarbell 2007), carotid bifurcation (Martorell et al. 2014) and aorta (Renner et al. 2012; Morbiducci et al. 2007), have been recently reported by our group at the blood–brain barrier (Garcia-Polite et al. 2016). To revert these changes, the medical device industry has developed hundreds of inventions, with percutaneous interventions such as stents and/or grafts becoming almost a commodity in patient care (Martorell et al. 2012). These devices modify permanently the structure of the vessel typically to allow blood circulation or modify blood behavior, focusing on a rather local effect. We and others have, however, proved that local modifications of blood flow have not only local but also downstream effects on the vasculature (He et al. 2015; Richter and Edelman 2006; Richter et al. 2004), which could be predicted and calculated (Martorell et al. 2014; Grigioni et al. 2005). Based on the knowledge that local modifications in flow could affect the downstream vasculature, we have developed the theory that by locally damping arterial pulses at the carotid bifurcation we could prevent fluidodynamic damage on the blood brain barrier. The mathematical work developed in this manuscript is the first step toward designing a graft that could damp pressure waves in the cerebrovascular system.

Several materials with medical applications, like polydimethylsiloxane (PDMS), popularly called silicone or polytetrafluoroethylene (PTFE), popularly called Teflon, show viscoelastic behavior (Calvo Aguilar 2013; Mahomed et al. 2015). These materials are inert and non-degradable, but their mechanical properties are very different to those of healthy arteries. PTFE and especially PDMS (Mahomed et al. 2015) are significantly stiffer than arteries (Clough et al. 2015; Bijnens et al. 2011) and have less viscous component (Mahata et al. 2006). However, to develop our mathematical model, we chose performing hysteresis tests on PDMS, which is easier to characterize. Our experiments showed significant energy absorption, which was then fitted to our mathematical model. Thanks to that, we have now a better understanding on the damping of a pulse along a straight pipe. This damping is exclusively due to pipe walls expansion, with minor elastic recovery as shown in our hysteresis tests. The simulations performed after material model adjustment showed that the Zener model, but not the Maxwell model, can be used to predict pulse attenuation in medical materials such as PDMS. We then estimated which combinations of material properties defined by the Zener model parameters can achieve a certain pulse attenuation for a given cylindrical pipe, obtaining an isosurface that depicts the required combination.

This manuscript studies pulse attenuation along a prosthesis of an eligible viscoelastic material. The mathematical expressions developed here suit two different purposes. On the one hand, one can now predict pulse attenuation by means of a cylinder made of a particular material. On the other hand, reverse engineering can be used to determine which characteristics a certain material should possess in order to reach a given pulse attenuation. We have tried which combinations of material properties are able to deliver a certain attenuation for a given pipe geometry. In the carotid device that we propose, we can predict an attenuation of up to 5% in a 200-mm-long cylinder using our mathematical method. This attenuation may seem mild, but our preliminary simulations have shown a significant decrease in shear stress at the blood–brain barrier thanks to this pressure damping. Further *in vitro* experiments will validate our initial estimations.

This is the first time, to the best of our knowledge, that a mathematical model for pulse attenuation including viscoelasticity along a cylindrical pipe has been studied using the Maxwell or the Zener material models. No definitive constitutive material model has been defined for blood vessels (Fung et al. 1979; Holzapfel et al. 2000; Valdez-Jasso et al. 2009; Masson et al. 2011; Sokolis 2013; Smoljkić et al. 2015; Wang et al. 2016; Holzapfel and Ogden 2010; Schulze-Bauer and Holzapfel 2003; Stålhand 2009). Recent works consider a viscoelastic Voigt (or Kelvin–Voigt) model for the vessel (Alastruey et al. 2012; Wang et al. 2015; Valdez-Jasso et al. 2009). The Kelvin–Voigt model cannot, however, represent the behavior of materials that show permanent deformation. Other models lead to non-isotropic and nonlinear models, which are different to those that fit materials for medical applications. Understanding fluid dynamics through blood vessels is a topic of outmost importance (Martorell et al. 2014; Garcia-Polite et al. 2016; Morbiducci et al. 2007; Assemat et al. 2014; Frank et al. 2002; García-Herrera and Celentano 2013; Boileau et al. 2015; Blanco et al. 2014). 3D calculations are the cornerstone to fully characterize blood flow behavior, and the fluid–solid coupled problem is nowadays one of the most exciting challenges in computational fluid dynamics (Chen et al. 2016; Cebral et al. 2015; Moireau et al. 2012). These models assume solid walls, elastic walls or more complex models. Windkessel models reduce the flow resistance of a vascular assembly to one point (Tsanas et al. 2009). Finally, 1D simulations, which have improved exponentially in recent years, usually assume elastic behavior of vessel walls. Despite their limitations in terms of spatial resolution, they have exponentially lower computational costs and can be a powerful tool to guide and constraint further calculations.

We have chosen a 1D approach for our model to minimize computational costs. Our model possesses a unique simplification that allows for easy calculations, if the material properties criteria are fulfilled. Instead of numerically estimating a solution for a third-order partial derivatives equation, we solve a simple analytical equation along a certain distance, for a certain time. A recent work (Pellicer and Solà-Morales in press) states a set of properties of the solutions to this equation by means of spectral analysis. The interested reader can find there the general statements about orthogonality and completeness of the eigenfunctions of a more general formulation. Using our simplifications, the attenuation achieved by a cylinder of a given material becomes a transfer function problem, which can be reduced to a pointwise resistive element.

Due to its simplicity, we are aware that our model has limitations. First, as already indicated, the model does not consider the effects of blood viscosity and the effect of stress in the longitudinal direction. In terms of wave propagation study, our model is still limited to an isolated pulse along a straight pipe, without considering bifurcations or arterial curvature. In this sense, the common carotid artery is one the straightest and flat vessels in the human body, and the device we would design would be a non-compliant cylindrical graft. Our model does not include the reflexive waves coming from arterial corners and bifurcations, which could indeed interact with the forward pulse wave. The overlapping of pressure waves can have multiple contrary effects as a function of the downstream geometry. Further research could help us reaching additional damping via wave superposition. As explained earlier, our model is also limited to a certain range of material properties. When the multiplication of the square wave velocity by the square time constant $\tau$ is inferior to 1 m$^{2}$, our model is unable to fit a simple exponential decay. This does not mean that no materials can achieve the desired attenuation, but it means that our simplified equations cannot predict this attenuation anymore. In those cases, the third-order partial derivatives equation must be solved numerically.

## Conclusions

We have presented a study of the decay of a pressure pulse propagating along a pipe made of a viscoelastic material. The Maxwell material model and the Zener model have been studied in this scenario, representative of a vascular prosthesis like a graft implanted in a blood vessel.

For the Maxwell material model, we have obtained an expression of exponential decay (46,47). For the Zener model, we have obtained an expression (46,48) that can be useful only for a range of cases. For relatively high values of the viscous coefficient, the steepest part of the pulse is damped quickly, leaving a smooth wave that slowly decays.

This work opens the window to better design vascular prosthesis made of viscoelastic materials for medical applications. In particular, it could lead to vascular devices able to modify pressure waves that jeopardize organs and structures sensitive to pressure waves like the blood–brain barrier.

## Electronic supplementary material

Below is the link to the electronic supplementary material.

Supplemental Video 1. Animated gif of a finite elements simulation where a tube section is flattened and consequently exposed to tension cycles at 50Hz.
