# Supplementary figures and images for: Arterial pulse attenuation prediction using the decaying rate of a pressure wave in a viscoelastic material model
Source: Biomech Model Mechanobiol. 2017 Nov 22;17(2):589–603. doi: 10.1007/s10237-017-0980-9 (PMC5845065; doi:10.1007/s10237-017-0980-9)

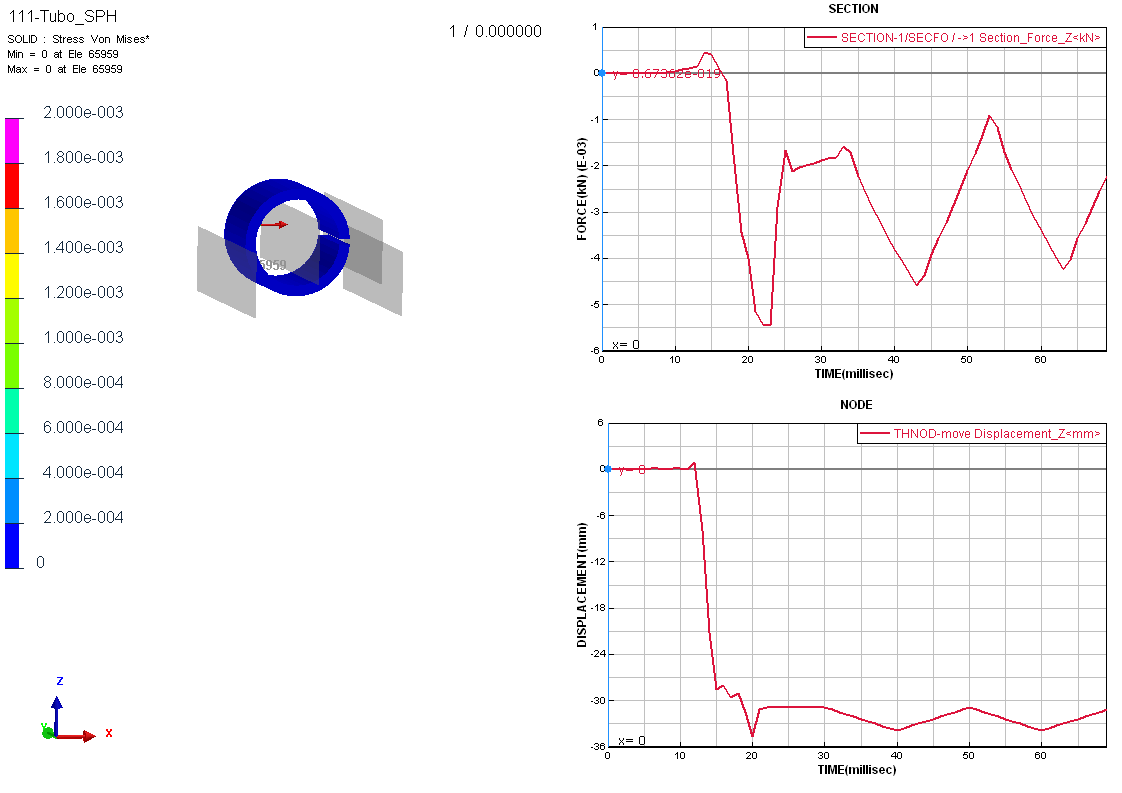

Supplement: Supplementary file 1 — Supplemental Video 1. Animated gif of a finite elements simulation where a tube section is flattened and consequently exposed to tension cycles at 50Hz. [file 10237_2017_980_MOESM1_ESM.gif]
